# Resource-Oriented Case Management to Implement Recommendations for Patients With Chronic Pain and Frequent Use of Analgesics in General Practices (Project RELIEF): Protocol for a Single-Arm Exploratory Feasibility Study

**DOI:** 10.2196/66335

**Published:** 2025-04-15

**Authors:** Regina Poß-Doering, Sabrina Brinkmöller, Alexandra Balzer, Viktoria Sophie Wurmbach, Cinara Paul, Regina Stolz, Marco Richard Zugaj, Jonas Tesarz, Michel Wensing, Cornelia Straßner

**Affiliations:** 1 Department of Primary Care and Health Services Research Medical Faculty, Heidelberg University University Hospital Heidelberg Heidelberg Germany; 2 Institute of Medical Biometrics Medical Faculty, University Heidelberg University Hospital Heidelberg Heidelberg Germany; 3 Department of Clinical Pharmacology and Pharmacoepidemiology University Hospital Heidelberg Heidelberg Germany; 4 Clinic of General Internal Medicine and Psychosomatics University Hospital Heidelberg Heidelberg Germany; 5 Institute for General Practice and Interprofesional Care University Hospital Tübingen Tübingen Germany; 6 Clinic of Anesthesiology, Section for Pain Medicine University Hospital Heidelberg Heidelberg Germany

**Keywords:** chronic noncancer pain, case management, primary care, general practice resource-oriented, chronic pain, analgesics, pharmacological treatment, pain medications, holistic approach, feasibility, single-arm, exploratory, pilot study, screening, questionnaire, survey, protocol

## Abstract

**Background:**

Chronic noncancer pain (CNCP) is a frequent reason for counseling in general practice. Current German guidelines emphasize its biopsychosocial etiology and the importance of self-care and nonpharmacological treatment strategies such as education, physical and social activity, and psychological approaches. Comprehensive assessments are necessary to individualize treatment maximally and monitor appropriate use of pain medication. General practitioners face many challenges in implementing holistic pain management, which considers biological, psychological, and social aspects. In project RELIEF (resource-oriented case management to implement recommendations for patients with chronic pain and frequent use of analgesics in general practices), a case management program was developed to facilitate implementation of guideline recommendations on pain management regarding medical assessment and monitoring, patient and practice team education, promotion of self-care strategies, and rational pharmacotherapy.

**Objective:**

We evaluated the feasibility of the intervention and study procedures before applying them in a larger cluster randomized controlled trial. Our secondary objective is to estimate potential effects of the complex intervention.

**Methods:**

A single-arm trial with general practices and patients with CNCP and analgesics use will be conducted, accompanied by a mixed methods process evaluation. The intervention comprises 5 components, including software-supported medical pain history, 3 scheduled structured appointments, e-learning on CNCP for general practitioners and medical assistants, educational material for patients, toolbox with information on (regional) resources for patients and practice teams. Participating practices will be located in the federal state of Baden-Württemberg, Germany, and will recruit eligible patients (adults with CNCP for more than 3 months, with at least moderate pain-related disability, permanent or on-demand use of analgesics or co-analgesics in the previous 4 weeks, and practice team assessed ability to participate actively in the trial). A questionnaire given to the first 150 adult patients entering the practice in February 2025 will help screen eligible patients. The primary objective will be measured by a set of predefined indicators. The key secondary outcome is pain-related disability measured by the Pain Disability Index German version. All participants will be asked to participate in the process evaluation. Outcome evaluation data will be gathered by paper-based and digitally provided questionnaires to be completed by participants. Process evaluation data will be gathered in surveys and a qualitative study. Descriptive analyses will be performed.

**Results:**

Recruitment occurred between October and December 2024. Targeted sample size was 6 practices and 50 patients. The intervention period will be February-June 2025. It is expected that eligible patients will benefit from the intervention and that improved medication management and intensified use of nonpharmacological treatment strategies will reduce pain-related disabilities and other patient-reported outcomes.

**Conclusions:**

This study will provide valuable information regarding feasibility and potential effects before testing the intervention in a confirmatory cluster randomized controlled trial.

**Trial Registration:**

German Clinical Trials Register DRKS00034831; https://www.drks.de/search/de/trial/DRKS00034831

**International Registered Report Identifier (IRRID):**

PRR1-10.2196/66335

## Introduction

### Background

About 20% of the patients in German general practices are affected by chronic pain [1], defined as pain that persists for more than 3 months or reoccurs [2]. Classification systems distinguish between chronic primary pain which cannot be explained by a detectable tissue damage (eg, fibromyalgia, unspecific low back, and psychosomatic pain disorders) and chronic secondary pain which is likely to have been caused at least initially by an organ or tissue damage (eg, degenerative or inflammatory diseases of the joints or spine and nerve damages) [2]. While cancer-related pain has characteristics of continuous or intermittent pain [3], chronic noncancer pain (CNCP) comprises any painful condition not associated with malignant disease and persisting for at least 3 months [4]. It interferes with activities of daily life and has a negative impact on quality of life and physical function [5]. CNCP is considered to be a major public health problem and one of the most common reasons why patients seek medical care [6,7]. The understanding of chronic pain pathogenesis has become more differentiated in recent years and there is consensus that chronic pain is always maintained or influenced by an interaction of biological, psychological, and social factors. Furthermore, psychological comorbidities such as depression, anxiety, or posttraumatic stress disorder are frequently associated with chronic pain [8]. Depending on which factors or comorbidities prevail, different treatment strategies are effective. Thus, comprehensive medical pain assessment is necessary in order to individualize treatment as best as possible.

Guidelines emphasize the importance of holistic pain management and nonpharmacological and noninvasive treatment strategies such as education, physical activity, social activity and support, relaxation techniques, or cognitive behavioral therapy. Analgesics should only be used temporarily and supportively until nonmedical treatments show an effect [9]. However, about two thirds of patients with chronic pain take analgesics [10] which may have severe adverse effects. Particularly alarming is the high percentage of patients taking nonsteroidal anti-inflammatory drugs (NSAID) which are also available as over-the-counter drugs. In a large telephone survey, 72% of German respondents with chronic pain stated using nonprescribed NSAID [10]. If taken on a long-term basis, NSAID may lead to renal insufficiency, gastrointestinal damages and cardiovascular events. Furthermore, the rise of opioid prescriptions for CNCP is observed with concern in Germany, even though there are no signs for an opioid epidemic [11]. The majority of opioid prescriptions are issued for CNCP, although it is well known that opioids are frequently not or little effective in this indication and associated with a high risk for adverse events such as obstipation, falls, cognitive impairments, or addiction [12]. If analgesics are applied, it is vital to perform thorough medication management and to adhere to monitoring recommendations. The German guideline for long-term use of opioids in chronic noncancer pain (LONTS) gives clear recommendations for safe opioid management [13]. However, adherence to guidelines might be impacted by factors such as personal attitudes, preferences, and experiences [14] and General practitioners (GP) might be less likely than other specialists to follow a guideline since they favor their own experience [15,16], or might not be aware of guidelines. Suboptimal pain management does not only result in unnecessary suffering of the affected patients but also in high costs for the health care system: patients with pain-related disabilities have a 6-fold higher rate of sick leaves and 4.5-fold higher rate of physician visits [17].

In Germany, patients with CNCP are mainly treated in ambulatory care by GPs and specialists such as orthopedists, neurologists, or rheumatologists. Only 10% have ever seen a pain specialist or received multimodal pain therapy [18]. GPs frequently know their patients and the patient’s family for many years. They often act as coordinators of care and are frequently the main prescribers of all medications. Therefore, they play a crucial role in the care of patients with chronic pain. In spite of these good preconditions, it remains challenging for GPs to implement structured and holistic pain management in their daily practice [9].

Within the project RELIEF (Resource-oriented case management to implement recommendations for patients with chronic pain and frequent use of analgesics in general practices), a case management program was developed to support GP in implementing guideline-based pain management. The program focusses on patients with CNCP and 4 essential areas of pain management: medical assessment and monitoring, practice team and patient education, self-care, and rational pharmacotherapy.

### Overall Aim of the Study

The main objective of this pilot study is to test the developed program in a small number of practices with a small number of patients to assess the feasibility of the developed intervention components (eg, assessment and e-learning) and methods used for recruitment (eg, screening process) and data collection. Thus, a control group (no intervention) will not be involved. The evaluation of the program’s potential effectiveness and data generation on the prevalence of the programs’ target group in German general practices are secondary objectives. Based on findings of this study, the program and evaluation concept will be adapted where applicable and applied in a subsequent confirmatory cluster randomized controlled trial (not described in this protocol).

## Methods

### Study Design

**Figure 1:**
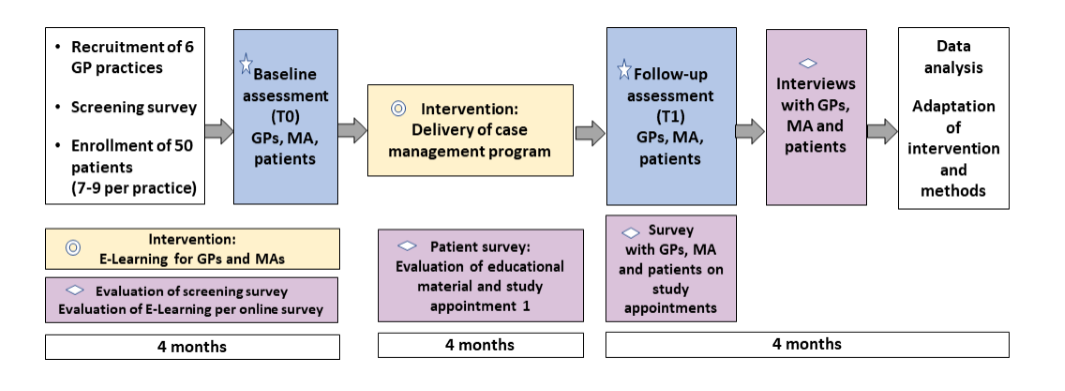
The design of the pilot study in RELIEF. Yellow box (circles): intervention components; blue box (star): outcome evaluation; purple box (diamond): process evaluation. GP: general practitioner; MA: medical assistant; RELIEF: resource-oriented case management to implement recommendations for patients with chronic pain and frequent use of analgesics in general practices.

A single-arm, exploratory pilot study with accompanying process evaluation will be conducted to pilot the case management program. Conducting a pilot study provides a good opportunity to assess feasibility of a full-scale study and can be considered an essential prerequisite to enhancing likelihood of success of the main study. Pilot studies should be well designed with clear feasibility objectives, and explicit criteria for determining feasibility [19]. Figure 1 details the design for the RELIEF pilot study.

### Intervention

The RELIEF intervention consists of five key components: (1) medical pain assessment and monitoring using a module specifically developed for the RELIEF intervention to be applied within an established case management software (CareCockpit) [20], (2) 3 scheduled structured appointments with GPs and medical assistants (MA), (3) e-learning on chronic pain management for GPs and MA, (4) educational video- and paper-based material for patients, and (5) a toolbox with a collection of resources for pain management for patients and practice teams. GP and MA will receive financial compensation for their additional efforts related to participation in the study (€200 [approximately US $216] per GP, €400 [approximately US $432] per MA) and number of included patients (€80 [approximately US $86] per included patient). GPs can apply for a total of 8 continuing medical education points upon completing the e-learning modules.

**Figure 2:**
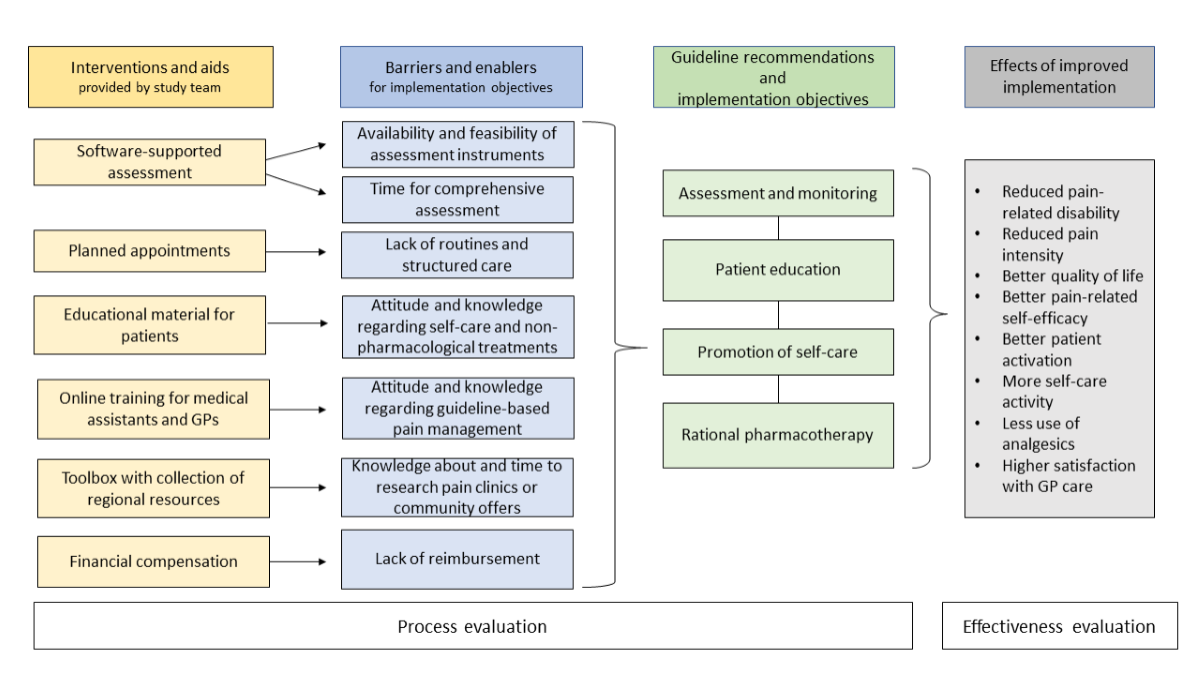
The assumed effect mechanism of the intervention. GP: general practitioner.

It is expected that patients participating in the case management program will benefit from the intervention. It is hypothesized that improved medication management and intensified use of nonpharmacological treatment strategies will result in a reduction in pain-intensity and pain-related disabilities, improved patient activation, more self-care activity and less use of analgesics. Figure 2 describes the assumed effect mechanism of the developed intervention.

It is assumed that adherence to guideline recommendations for CNCP by practice teams (green boxes) will improve if the intervention components (yellow boxes) effectively reduce identified barriers or make use of identified enablers respectively (blue boxes). Improved adherence to guideline recommendations will result in improved health outcomes or health behavior, respectively (gray box). Primary and secondary outcomes (gray box) focus on the effects of improved guideline adherence while adherence as well as assumed linkages between interventions and barriers are assessed within the scope of a comprehensive process evaluation.

After enrolling in the pilot study, patients will complete a pain assessment through a browser-based proprietary app available to study participants only (TeleVital app linked to CareCockpit) at home on their own digital device (smartphone, tablet, computer, etc). The assessment focusses on various aspects of pain and mental comorbidities. Within 2 weeks, patients will have a scheduled structured appointment (study appointment 1) with their GP during which the patients’ specifications in the assessment will be discussed and complemented by an open pain history taking. For this purpose, GPs or medical assistants will perform a manual import of the assessment data into the software CareCockpit, an established case management software currently used by about 800 general practices in Baden-Württemberg, Germany, for the case management program PraCMan [20]. This is a care model for patients with multimorbidity insured by the statutory health insurance AOK Baden-Württemberg. The data import will only be started when the patient is present in the practice to ensure that GPs are able to react immediately on critical information such as severe depression. Practice teams will receive the data in 2 versions: the original version with all items and patients’ responses and, in addition, a summary of the assessment with automatically calculated scores.

At the end of study appointment 1, educational material on chronic pain (a booklet and links to educational videos tailored to patient’s self-efficacy, to be used as often as patients want, and at their own speed) will be handed out to patients and the date for study appointment 2 will be set for about 2-4 weeks later. During study appointment 2, patients and GPs or patients and medical assistants will reflect on the educational material provided and agree on treatment goals related to daily activities (eg, to be able to do some gardening) and self-care activities (eg, physical activity or relaxation techniques). If necessary, medication is prescribed and therapies outside the general practice are initiated (eg, physiotherapy, multimodal pain therapy, psychotherapy, and rehabilitation), and monitoring activities (diagnostics, amendments to therapy plan, discussion of possible adverse effects, pain management, medication, self-care, etc) are planned. A treatment plan containing all this information will be issued by the responsible GP through the CareCockpit software and the date for study appointment 3 will be set for 4-6 weeks later. During study appointment 3, practice teams will check whether activities could be applied as planned. If necessary, the treatment plan will be adapted. The activities performed during study appointments 1-3 will briefly be documented by the practice teams through a checklist in the CareCockpit module. The intervention period will end with a follow-up assessment 4 weeks after study appointment 3.

A webinar will be offered by the study team to go over study organization and measures with all participating practice teams. Participating GPs and MAs will complete an e-learning on chronic pain management with 4 modules covering the pathogenesis of chronic pain, self-care activities (relaxation technics, physical activity, and topical applications), analgesics and interprofessional and interdisciplinary pain therapy. The e-learning is expected to be completed before the first patient receives study appointment 1. A website with a toolbox containing useful links and information on chronic pain as well as a collection of regional resources (eg, hospitals providing multimodal pain therapy and counseling centers etc.) will be provided by the study team. Table 1 summarizes the planned course of the RELIEF case management program.

**Table 1:** The course of the case management program in the RELIEF^a^ pilot study.

Who		Where	What
**Week 0**			
	MA^b^	Practice	Enrollment into study
	Patient	At home	Structured pain assessment through TeleVital appT0 baseline assessment through a paper-based questionnaire
**Week 2**			
	GP^c^	Practice	Study appointment 1: Discussion of the results of the structured pain assessment Open pain history taking Patient receives educational material
	Patient	At home	Reading educational material or watching educational videos, respectively
**Week 5 to 6**			
	MA MA or patient GP	Practice	Study appointment 2: Reflection on educational material Agreement on treatment goals and self-care activities Treatment plan is issued
	Patient	At home	Applies treatment plan
**Weeks 10 to 12**			
	MA GP	Practice	Study appointment 3: Monitoring if treatment plan and self-care could be applied as planned; if necessary, adaption of the treatment plan
	Patient	At home	Applies (adapted) treatment plan
**Week 16**			
	Patient	At home	T1 follow-up assessment; end of pilot study

^a^RELIEF: resource-oriented case management to implement recommendations for patients with chronic pain and frequent use of analgesics in general practices.

^b^MA: medical assistant.

^c^GP: general practitioner.

### Recruitment

General practices will be recruited through known contacts such as the established practice network of about 800 practices that use a particular case management system for chronically ill patients (PraCMan) [21] and a network of teaching and research practices affiliated with the Department of Primary care and Health Services Research, University Hospital Heidelberg. The target is to include 6 practices. Based on previous experiences, it can be assumed that about 10% of the practices approached are interested in participating in research projects. Therefore, 60 practices in a predefined region will be randomly drawn and an invitation to participate in the pilot study will be sent to them by postal or electronic mail together with a declaration of interest form. Practices that declare interest in participation will receive written information about study aims and procedures. In case the recruitment target is not met, another random sample will be drawn.

To identify eligible patients and to gather information on the prevalence of the target group in primary care, a screening survey will be conducted (see Data Collection and Outcomes section below). Eligible for participation will be adult patients with CNCP for more than 3 months, with at least moderate pain-related disability (minimum 4 points on a scale from 0-10), permanent or on-demand use of analgesics and co-analgesics in the previous 4 weeks, and practice team assessed ability to participate actively in the pilot study (sufficient cognitive abilities and internet access). Patients with cancer pain or in palliative care will be excluded. Recruitment target for each practice is 7-9 patients. If more than 9 patients agree to participate in the piloting, the practice team will select 7-9 patients who are according to their assessment likely to benefit from the program. Specific reasons for their choices will be explored in the process evaluation.

### Data Collection and Outcomes

For the screening survey, each participating practice will hand out a screening questionnaire to the first 150 adult patients entering the practice from a defined date on. The practice team will collect the questionnaires, check eligibility with a provided template and invite all patients meeting the inclusion criteria to participate in the pilot study by handing out the study information material. The practice team will add information regarding reasons for nonparticipation, known CNCP diagnosis, known use of analgesics, and prescription of analgesics during the last month on the screening questionnaires of all patients meeting the inclusion criteria (regardless of whether they agree to participate in the study or not) by checking the patient file. The practice team will deidentify all questionnaires (also of patients without chronic pain) by cutting the lines for name and birthdate and send them to the study center for evaluation purposes regarding prevalence of patients in a primary care setting who meet the inclusion criteria.

Data collection related to medical pain history (used by practice teams only, not by researchers) comprises the following steps: patients receive a weblink from their GP practice leading to a browser-based app called TeleVital and complete the assessment through the app on their own digital device. TeleVital is a proprietary development of the Department of Primary Care and Health Services Research, University Hospital Heidelberg, and facilitates structured data collection in studies which use the CareCockpit. Data generated in the TeleVital app data will be stored on a secure server located at the University Hospital Heidelberg, Germany until it is transferred to the CareCockpit software installed in the GP practice. Data import will be initiated manually by the practice team at the next patient contact. Thus, practice teams will receive the assessment data only when the patient is present in the practice. Practice teams will use the assessment data for the purpose of diagnostics and treatment. It contains information on pain history, pain characteristics, use of analgesics, use on nonpharmacological measures, treatment targets, and items from validated screening questionnaires such as the Pain Detect questionnaire on neuropathic pain [22] and on mental comorbidities such as posttraumatic stress disorder [23].

Data collection for the outcome evaluation comprises the following steps: primary objective of this pilot study is to assess the case management’s feasibility, measured by a set of predefined feasibility indicators. Regarding the intervention components, feasibility indicators refer to the software-supported medical pain history, study appointments, educational material for patients, and e-learning for GPs and MA. Feasibility of study procedures will be measured for the patient recruitment and enrollment process, completion of T0 and T1 questionnaires and drop-out rate. To facilitate this assessment, transfer of partial documentation from the CareCockpit to the study center will be used either electronically or paper-based, depending on preference and available resources in the participating practices. Table 2 describes the feasibility indicators in relation to the intervention components, and Table 3 details the feasibility indicators for study procedures.

**Table 2:** The feasibility indicators for intervention components in the pilot study.

Interventions	Data source	Indicator	Rating
Software-supported medical pain history	CareCockpit data process evaluation	Percentage of patients with pain assessment transferred to practice computer	≥80%: feasibility given <80%, but reasons solvable by modification: feasibility likely <80% and reasons not solvable by modification: feasibility not given
Planned appointments	CareCockpit data	Percentage of patients who received all 3 study appointments	≥80%: feasibility given <80%, but reasons solvable by modification: feasibility likely <80% and reasons not solvable by modification: feasibility not given
Educational material for patients	Evaluation questionnaire and patient interviews	Percentage of patients who perceived the provided content relevant and comprehensible	≥80%: feasibility given <80%, but reasons solvable by modification: feasibility likely 80% and reasons not solvable by modification: feasibility not given
E-Learning for GPs and medical assistants	Evaluation questionnaire and general practitioner and medical assistant interviews	Percentage of health care professional who perceived the e-learning relevant and comprehensible.	≥ 80%: feasibility given <80%, but reasons solvable by modification: feasibility likely 80% and reasons not solvable by modification: feasibility not given
Toolbox	Survey and general practitioner, medical assistant, and patient interviews	Percentage of participants who perceived the toolbox as relevant	≥80%: Feasibility given <80%, but reasons solvable by modification: feasibility likely 80% and reasons not solvable by modification: feasibility not given

**Table 3:** The feasibility indicators for study procedures in the pilot study.

Study procedure and data source		Indicator	Rating
**Patient recruitment**			
	Screening questionnaire	Percentage of patients who agreed to participate in the screening survey	>80%: feasibility given <80%, reasons solvable by modification: feasibility likely <80% and reasons not solvable by modification: feasibility not given
	Screening questionnaire	Percentage of patients in screening survey meeting all inclusion criteria	>10%: feasibility given <10%, but reasons solvable by modification: feasibility likely <10% and reasons not solvable by modification: feasibility not given
	Screening questionnaire	Percentage of practices that enrolled 7-9 patients	>80%: feasibility given <80%, reasons solvable by modification: feasibility likely <80% and reasons not solvable by modification: feasibility not given
**Outcome evaluation**			
	T0 and T1 questionnaires	Percentage of patients who completed T0 and T1 questionnaires	>80%: feasibility given <80%, reasons solvable by modification: feasibility likely 80% and reasons not solvable by modification: feasibility not given
	General practitioner report	Patient drop-out rate	<20%: feasibility given >20%, but reasons solvable by modification: feasibility likely >20% and reasons not solvable by modification: feasibility not given

A range of secondary outcomes will be determined to gather information on potential effects of the program. All secondary outcomes are participant-reported and will be collected before (T0) and after (T1) the intervention by paper-based questionnaires which participants will complete at home and send directly to the study center.

Outcome measures on patient level comprise the German versions of the following validated instruments: Pain Disability Index German version (PDI-G) [24] (key secondary outcome), Patient Activation Measure [25], Short Form 12 Health Survey scale for health related quality of life [26], De-Jong-Gierveld loneliness scale [27], Pain Self-Efficacy Questionnaire German version [28], Avoidance-Endurance Fast Screening instrument [29], Pain-Catastrophising Scale [30], selected items of the European Project on Patient Evaluation of General Practice Care (EUROPEP) questionnaire on evaluation of GP care [31,32], and pain intensity (numeric analogue scale). Further study-specific items will be used to assess the use of self-care such as physical activity (3 items), relaxation techniques (1 item) and use of topical applications (1 item) and patients will be asked to document their pain medication (over-the-counter and prescribed). Secondary outcomes on GP and MA level refer to quality indicators for ambulatory pain management developed in the RELIEF project. Data for the process evaluation will be collected at various points in time as detailed in Figure 1.

During patient recruitment, practice teams will be asked to send the deidentified patient screening questionnaires to the study center for evaluation purposes. The e-learning for GPs and MA will be hosted on the platform Moodle on the server of the aQua Institute, Göttingen. After completion of the e-learning GPs and MA will be asked to fill in a short digitally provided survey to evaluate the training regarding aspects such as subjective knowledge increase, appropriateness of required time, appropriateness of didactical methods, and usability of the platform. The survey will be conducted through the survey tool Lime Survey hosted at a server of the University Hospital Heidelberg. Survey data will be linked through a pseudonym to the following meta-data gathered by the e-learning platform (e-learning completed or not completed, time needed to complete the e-learning (minutes), and number of logins necessary to complete the e-learning).

Patients will complete a paper-based pseudonymized questionnaire focusing on use and perceived usefulness of the provided educational material and experiences with study appointment 1. Patients will complete the questionnaire after going through the content provided and within 4 weeks after study appointment 1. At the end of the intervention period, patients will be asked to fill in a second questionnaire focusing on their experiences with study appointment 2 and 3 as part of the T1 follow-up survey.

All participating GPs and medical assistants will be invited to report their experiences during the pilot in a telephone interview. All patients will be invited to an interview after completing study visit 3. Depending on the response rate, a purposive sample of patients will be drawn. Written informed consent to participate will be obtained using separate information documents and agreement forms. Key questions on the interview guides will refer to intervention feasibility with regards to specified feasibility indicators, usefulness of provided material and tools, perceived effectiveness of the case and care management program, and suggestions for modifications from the perspective of health care providers and patients. Interviews will be recorded and transcribed verbatim. Transcripts will be pseudonymized and stored on secure servers at the Department of Primary Care, University Hospital Heidelberg. All audio files will be deleted after completion of data analysis.

### Data Analysis

A plan for the primary analysis will be finalized before data bank closure. All analysis will be performed in R (version 4.2.0 or higher; R Foundation for Statistical Computing) or in SPSS (version 28.0.1.0; IBM Corp) in a validated environment. The final analysis will be done as soon as the database has been declared to be complete and accurate and has been locked. Descriptive statistics will be provided to summarize demographics and baseline characteristics. In general, continuous variables will be described using number of observations, mean, SD, median, Q1, Q3, minimum, maximum, 95% CIs and, if existing, number of missing values at T0, T1 and for T0-T1 if appropriate. For categorical variables, absolute and relative frequencies will be given with missing values being reported as a separate category at T0 and T1. A CONSORT (Consolidated Standards of Reporting Trials) flow diagram will be created to display the progress of all participants through the trial. This includes the number of patients assessed for eligibility and the number of patients excluded because they did not meet inclusion criteria, declined to participate, or any other reason.

### Primary Outcome Analysis

For each objective of the intervention components and study procedures, a descriptive analysis will be performed to assess feasibility. For each objective, 95% CIs will be given based on the Wilson Score interval for binary measures [33]. Reasons for not fulfilling an indicator will be categorized and evaluated descriptively.

### Secondary Outcomes Analysis

As one of the aims of the study is to estimate the effect size, the 95% CI (2-sided) of the PDI-G percent change from baseline to T1 (T0-T1), which is planned to be the primary outcome in a subsequent cluster randomized trial and therefore of major interest in this pilot study, will be given. Only questionnaires with at least 6 out of 7 answered items will be considered. Missing items will not be imputed. Questionnaires with less than 6 answered items will not be considered.

As a sensitivity analysis, missing values of the PDI-G will be replaced on item level using multiple imputation based on predictive mean matching using the variables age, sex, center, and pain intensity at baseline as potential predictors. Missing scores and differences can then be calculated using the imputed items. Furthermore, a best- and worst-case scenario will be looked at, where missing values will be imputed by the highest and lowest observed value for T0 and T1 in the best case scenario and vice versa in the worst case scenario (lowest and highest observed value for T0 and T1, respectively). As another sensitivity analysis, a mixed linear regression model for the dependent variable PDI-G score at T1 will be performed including age, sex, and PDI-G at T0 as fixed effects and center as random effect. Descriptive analysis at item level and for the total number of pain-related patients at T0 and T1 will be done. Descriptive analysis of the item and score level will be done for all remaining secondary outcomes. For differences in scores between baseline and follow-up, 95% CIs will be reported. Missing values for secondary outcomes will not be imputed.

No sample size calculation was performed, as the main purpose of the pilot study is to investigate feasibility of the applied interventions and study procedures. Considering the exploratory nature of the pilot study, and based on experiences from previously conducted research, a total of 50 patients is considered sufficient to provide an initial estimate of the potential effect of the intervention measured by the PDI-G percent change from baseline to T1 (T0-T1), which is the key secondary endpoint. Assuming a SD of 13.7 and 15.4 score points [34] corresponding to a SD of 19.6% and 22% score points respectively, a sample size of 50 patients and a 2-sided 95% CI would yield the following: assumed SD 19.6: [x-5.6, x+5.6]; assumed SD 22: [x-6.3, x+6.3], where x is the point estimate of the PDI-G percent change from baseline to T1 (T0-T1).

### Analysis of Process Evaluation Data

Analysis of the qualitative data collected in the process evaluation will use an inductive approach based on the themes covered in the interview guide (including unintended effects). Data management will be done in MAXQDA (Verbi Software). Written surveys will be conducted digitally through the survey tool Lime Survey hosted on secure servers of the Heidelberg University. All quantitative survey data and data from free text fields will be analyzed descriptively using SPSS and visualized in Excel (Microsoft). In addition, data entered by the practice teams into the CareCockpit software to document activities performed during the study appointments will be transferred to the study center and analyzed within the scope of the process evaluation. All data will be deidentified before analysis. All data generated in the process evaluation will be triangulated for classification of intervention and program feasibility. Final assessment of feasibility will be performed by 2 researchers in a consensus process.

### Ethical Considerations

The pilot study in RELIEF received ethics approval from the Ethics Committee of the Medical Faculty at University Heidelberg, Germany (S-329/2024; June 05, 2024) and the state medical association of Baden-Württemberg (B-F-2024-057; July 02, 2024). The study will be conducted in accordance with the Declaration of Helsinki. All participants will give written consent before participation.

## Results

The recruitment for this pilot study started in October 2024 and was open until end of December 2024. The targeted sample size was 6 practices and 50 patients. The intervention period began in February 2025 and will run until June 2025. Findings are expected to provide an indication of potential patient benefits as well as feasibility of the interventions and study procedures. First evaluation results regarding potentially necessary adaptations of intervention components and study procedures are expected to be available in July 2025. Publication of findings and necessary adaptations are expected for the second and third quarters in 2025.

## Discussion

### Principal Findings

Findings in this exploratory pilot study will provide a clear indication regarding intervention feasibility and applied study procedures. It is expected that eligible patients will benefit from the intervention and that improved medication management and intensified use of nonpharmacological treatment strategies will result in a reduction of pain-related disabilities and other patient-reported outcomes. Potentially necessary adaptations of intervention components and study procedures will be finalized before testing the intervention in a randomized controlled trial which is planned to begin late in 2025. The evidence base regarding the chosen combination of intervention measures for patients with CNCP in German primary care is still limited. The expected effects appear to be plausible; however, they are not certain, so this pilot study can provide an important contribution.

The common belief that pain is a normal part of aging may explain why chronic pain is often underestimated and underreported [35]. Studies found that older adults tend to adopt a stoic attitude toward experiencing pain and prefer to use self-reliance–based coping strategies, though these may not always be effective [35,36]. A study in Italy explored coping strategies used by older adults to manage chronic pain and found that frequently coping self-statements, resting, task persistence, and guarding were described, while the least used strategies were relaxation and exercise or stretching [37]. The latter are clearly recommended in German guidelines [9]. Other studies also explored life experiences and needs of older adults with chronic pain and strategies they use to cope with and manage pain [38,39] or aimed to promote effective and tailored pain self-management interventions [36,40]. The RELIEF case management program contains innovative elements: GPs, MAs, and patients may benefit from the more structured care processes and patient activation which in turn can provide room and time for open discussion with patients on their needs. Meticulously obtained pain history for example, provides important information for pain assessment regarding onset and course, pain episodes, quality, intensity, activity impairment, and any stress factors in a patient’s personal life [41]. Some elements of the innovative program can be assigned to medical assistants, for example reflecting on the educational material or setting individual treatment targets with patients. Thus, MA will be more involved in CNCP care than usual, which may increase their competences, strengthen their role in the care process, and support sharing of the workload in team-based care [42].

Educational components will be used as an explicit treatment strategy anchored directly in the general practice setting. Care programs for disease management usually offer patient education outside of the GP practice which has the disadvantage of GPs being unfamiliar with the presented content which makes it difficult to refer to and pick it up in the course of treatment [9]. Combining therapies with delivering content remotely through the internet or mobile devices is increasingly used to promote and improve self-management of chronic conditions. And complement face-to-face pain treatments [43]. On the other hand, some elements of the RELIEF program may cause additional burden: patients will be asked to provide detailed information about their pain history, mental condition and personal goals and receive comprehensive information about chronic pain. This may provoke adverse effects such as negative, stressful emotions, yet also has the potential for reflection about perhaps still unused self-care potential and possible behavior adaptation. Participation in the case management program will require additional appointments and therefore be associated with an additional time burden for patients and practice teams alike. This pilot study will assess thoroughly whether the assumed effect mechanisms are plausible and whether the potential benefits outweigh potential harms.

### Strengths and Limitations

This study is a single-arm exploratory pilot study which leaves room for bias. Primary and secondary outcomes are based on self-reported data. In the multifaceted program, the potential impact of the various components might be difficult to separate. Strong aspects will be the program’s closeness to daily practice and the exploration of implementation outcomes in the accompanying process evaluation which adds value to this study. The analysis of components of the complex implementation program using predefined feasibility indicators contributes to the transparency of the implementation program.

### Dissemination Plan

After completing this feasibility study, findings will be disseminated through oral and poster presentations at scientific conferences as well as in a scientific article in a peer-reviewed journal.

### Conclusion

This study will provide valuable information regarding potentially necessary adaptations before applying the intervention in a confirmatory cluster randomized controlled trial.

## Appendix

Multimedia Appendix 1 SPIRIT checklist.

## Abbreviations

CNCP: chronic noncancer pain
CONSORT: Consolidated Standards of Reporting Trials
EUROPEP: European Project on Patient Evaluation of General Practice Care
GP: general practitioner
LONTS: German guideline for long-term use of opioids in chronic noncancer pain
MA: medical assistant
NSAID: nonsteroidal anti-inflammatory drug
PDI-G: Pain Disability Index German version
RELIEF: resource-oriented case management to implement recommendations for patients with chronic pain and frequent use of analgesics in general practices

## Data Availability

In line with the obtained ethics approval, all data sets that will be generated and analyzed during this pilot study and the process evaluation will not be publicly available due to data protection laws. They will be stored on secure servers at the Department of Primary Care and Health Services Research, University Hospital Heidelberg. Deidentified sets of these data can be made available by the corresponding author on reasonable request.
